# Disseminated Cryptococcosis in a Patient With Metastatic Prostate Cancer Who Died in the Coronavirus Disease 2019 (COVID-19) Outbreak

**DOI:** 10.7759/cureus.8254

**Published:** 2020-05-23

**Authors:** Matteo Passerini, Roberta Terzi, Marco Piscaglia, Simone Passerini, Stefania Piconi

**Affiliations:** 1 Infectious Disease, Luigi Sacco Hospital, University of Milan, Milan, ITA

**Keywords:** cryptococcosis, cryptococcus neoformans, opportunistic infection

## Abstract

We report the case of a 61-year-old patient with a history of prostate cancer affected by bone metastasis. He presented to our attention for ulcerous and necrotic cutaneous lesions unresponsive to antibiotics. The spread of cutaneous lesions and the onset of neurological symptoms suggested a cryptococcal disease, which was confirmed by lumbar puncture and cutaneous biopsy. We present the diagnostic and therapeutic approach to this case.

## Introduction

Cryptococcosis is an invasive fungal infection due to Cryptococcus neoformans or Cryptococcus gattii; the former is the principal pathogenic member of the genus and has a worldwide distribution [1].

Cryptococcosis is typical of immunocompromised patients, and among them historically HIV-positive patients, but there are some reports of presumed immunocompetent patients [2,3].

This disease can affect different organs: meningoencephalitis is the most described manifestation followed by pulmonary and cutaneous disease. The clinical presentation is heterogeneous, ranging from a mild disease to a life-threatening condition. It often presents with a subacute course with a delayed diagnosis, especially if a patient has various comorbidities.

## Case presentation

A 61-year-old male patient affected by stage IV prostate cancer with bone metastasis at diagnosis presented to our Wound Care Clinic complaining of ulcers on the right forearm that appeared two weeks prior following a referred domestic trauma.

During the physical examination, we observed two ulcers on the right forearm: a 6 cm x 4 cm proximal posterior ulcer covered by fibrin and a 2-cm proximal anterior ulcer with odorous exudate. There was no presence of crackles on palpation of the area surrounding the ulcers. Following a cutaneous swab, which resulted positive for unspecified yeast, the patient was admitted to our clinic.

Besides the aforementioned cancer disease, the patient’s medical history included hypertension, cholelithiasis, colon-sigma diverticulosis, and a recent discharge from another hospital for sepsis, bladder rupture, and multifocal pneumonia without microbiological evidence, which were treated successfully with piperacillin/tazobactam.

The patient’s pharmacological therapy consisted of bicalutamide, high-dose dexamethasone (4 mg a day for more than three months), oxycodone/naloxone, pregabalin, celecoxib, zoledronate, irbesartan, amlodipine, bisoprolol, proton pump inhibitors, and delorazepam.

**Figure 1:**
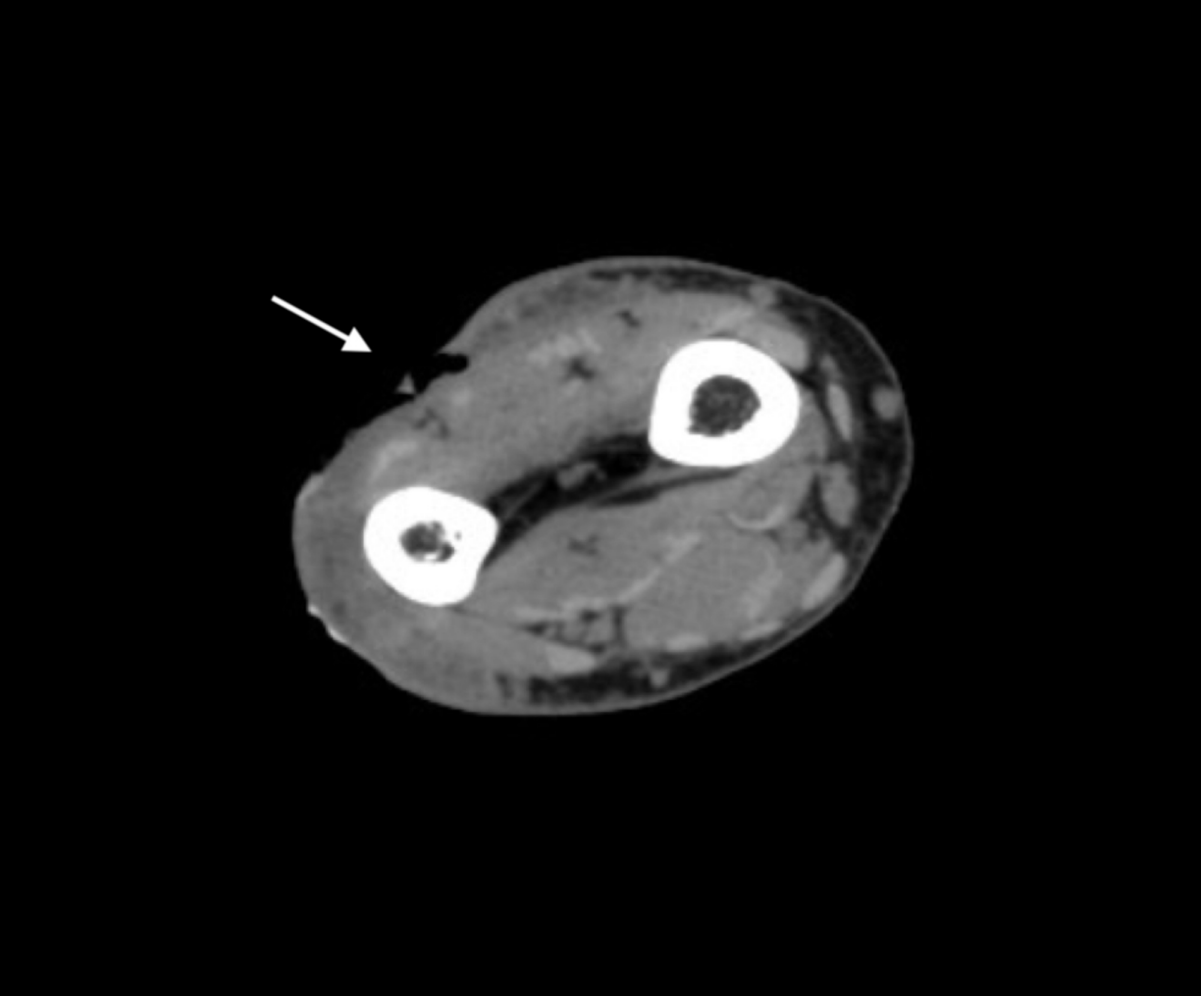
CT scan (axial view) of the right arm at the presentation Limited superficial aerial images (arrow) showing inhomogeneity of the subcutaneous fat.

CT scan of the right forearm revealed an extended inflammation of the dermis and hypodermis and excluded any abscesses or signs of necrotizing fasciitis (Figure 1). Blood tests revealed the presence of moderate inflammation with a CRP (C-reactive protein) of 24 mg/L WBC (white blood cell) count of 18.13 x 10^9^/L, creatinine of 0.56 mg/dL, and ALT (alanine aminotransferase) of 52 U/L.

At the time of admission, the patient was afebrile. For the cutaneous lesion, empiric therapy was started with piperacillin/tazobactam 4.5 g every eight hours along with advanced dressings determined by the wound care physician.

**Figure 2:**
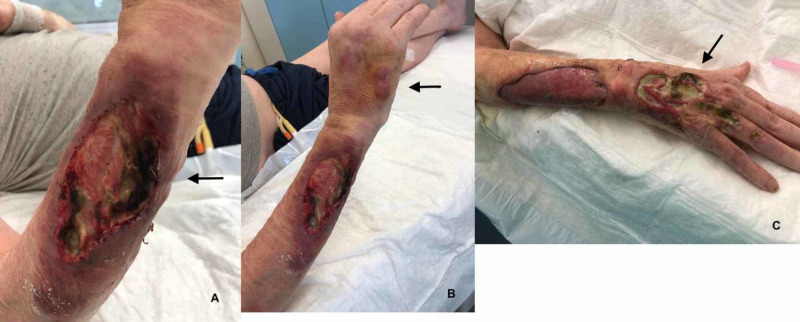
Progression of the cutaneous lesions Initially, the lesions were only on the forearm (A) and then spread to the hand (red arrows) as papules (B), which became ulcers (C). Note the progressive extension of the initial wound.

In the following days, we observed an extended loss of organic substance (Figure 2A) with a progressive expansion of the ulcers (Figures 2B, 2C) despite the surgical curettage performed by the plastic surgeon and the advanced dressings performed by the wound care physician.

NNegative-pressure wound therapy was started without success, and the cultured foam samples isolated methicillin-resistant Staphylococcus aureus (Panton-Valentine negative). Therefore, we initiated vancomycin 1 g every 12 hours plus meropenem 1 g every 8 hours followed by a course of daptomycin 600 mg every 24 hours for the isolation of vancomycin-resistant Enterococcus in the rectal swab.

**Figure 3:**
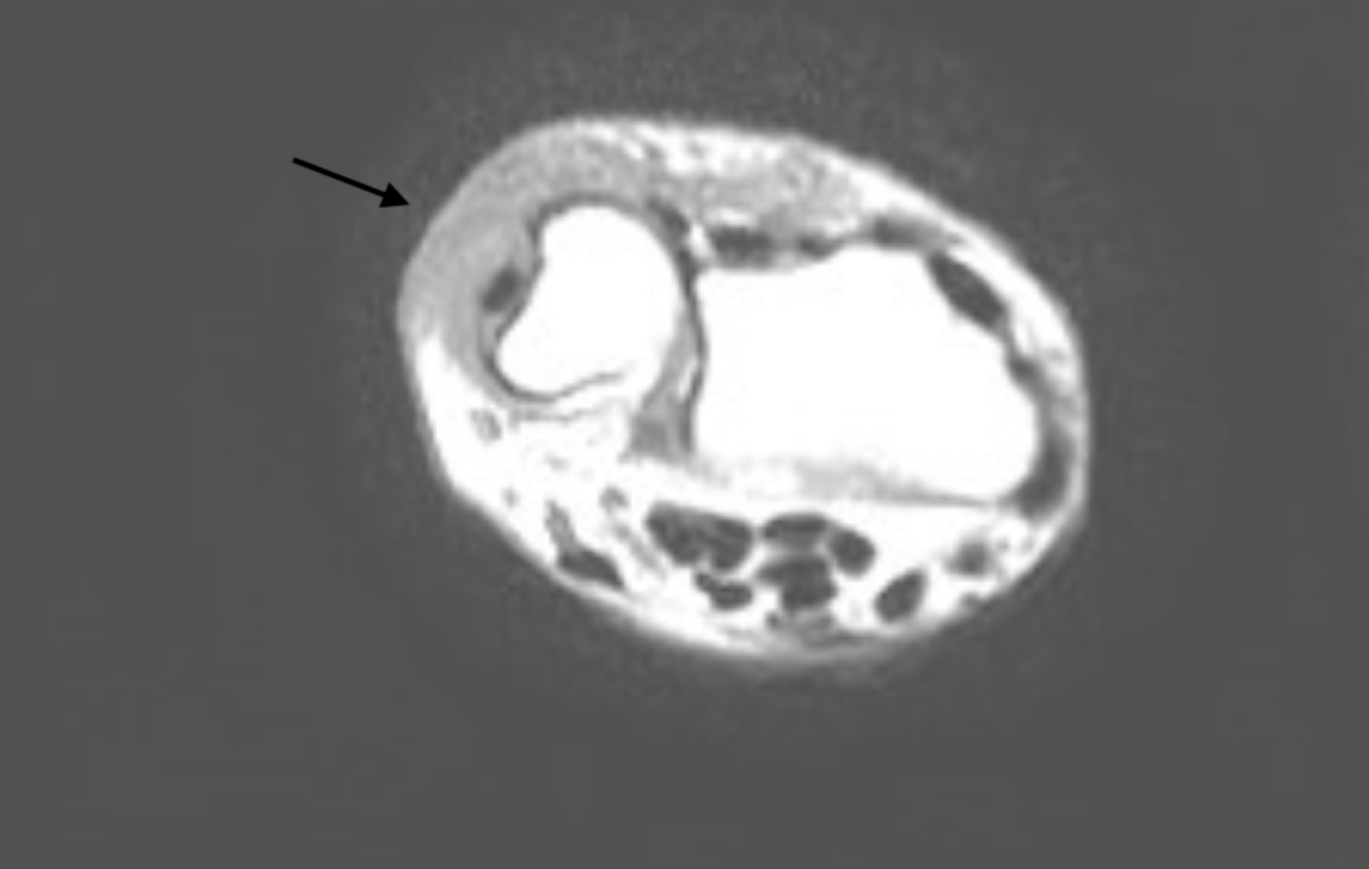
MRI (T1-weighted) of the right arm MRI did not show signs of osteomyelitis, but a signal alteration compatible with corpuscle fluid collection (arrow) could be seen, suggesting a florid activity of infectious inflammatory disease.

Despite the aforementioned therapy, the cutaneous lesion did not improve but instead advanced. Two new cutaneous lesions manifested on the right hand and left thigh. Both were red and non-tender papules and very similar to the initial presentation as reported by the patient prior to admission (Figure 2B). Subsequently, echocardiography excluded infectious endocarditis (Video 1) and an MRI of the right arm excluded osteomyelitis (Figure 3). To note, during hospitalization, blood tests showed only a minimal increase in the inflammation markers.

**Video 1:** Echocardiography showing no signs of infective endocarditis

In the following days, the patient complained of frequent episodes of confusion and tremor associated with an increasing headache. CT of the brain was negative, electroencephalogram showed a diffuse pattern of encephalopathic suffering, and the neurological examination performed by a specialist showed metabolic encephalopathy.

Due to the association between the failure of broad-spectrum antibiotics and the onset of neurological symptoms, especially headache, we performed a lumbar puncture (Table 1) that isolated Cryptococcus neoformans. The cryptococcal antigen on serum was positive, and a cutaneous biopsy of the ulcers confirmed the presence of the yeast. Therefore, we started induction antifungal therapy with amphotericin B plus flucytosine and switched to consolidation therapy with fluconazole 400 mg following a repeat of the lumbar puncture (Table 1) after 16 days.

**Table 1:** Results of the two lumbar punctures

	First lumbar puncture	Lumbar puncture after 16 days
Cryptococcal antigen	8,192	1,024
India ink	Positive	Negative
Cultures	Positive	Negative
Proteins	3,014 mg/L	2,356 mg/L
Glucose	6 mg/dL	52 mg/dL
Leukocytes	0.029 x 10^9^/L	0.16 x 10^9^/L

The immunological studies showed a profound cellular and humoral immunosuppression: CD4+ count of 0.123 x 103/mL, CD8+ count of 134 x 103/mL, CD4/CD8 count of 0.91, IgG of 4.18 g/L (normal range: 7.00-16.00), IgA of 0.79 g/L (normal range: 0.70-4.00), and IgM of 0.41 g/L (normal range: 0.40-2.30). The HIV test was negative.

**Figure 4:**
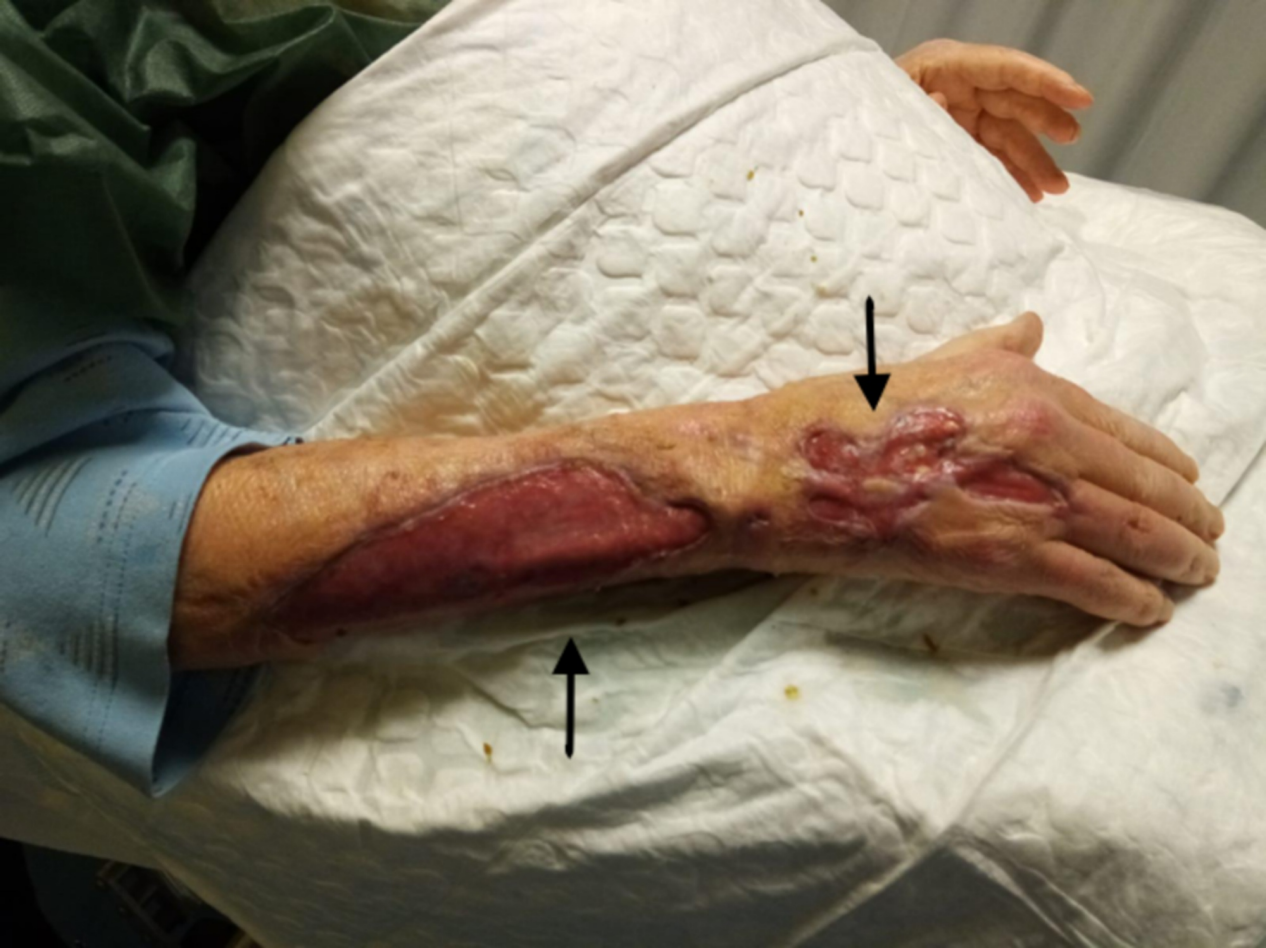
The cutaneous lesions after the first plastic surgery

**Figure 5:**
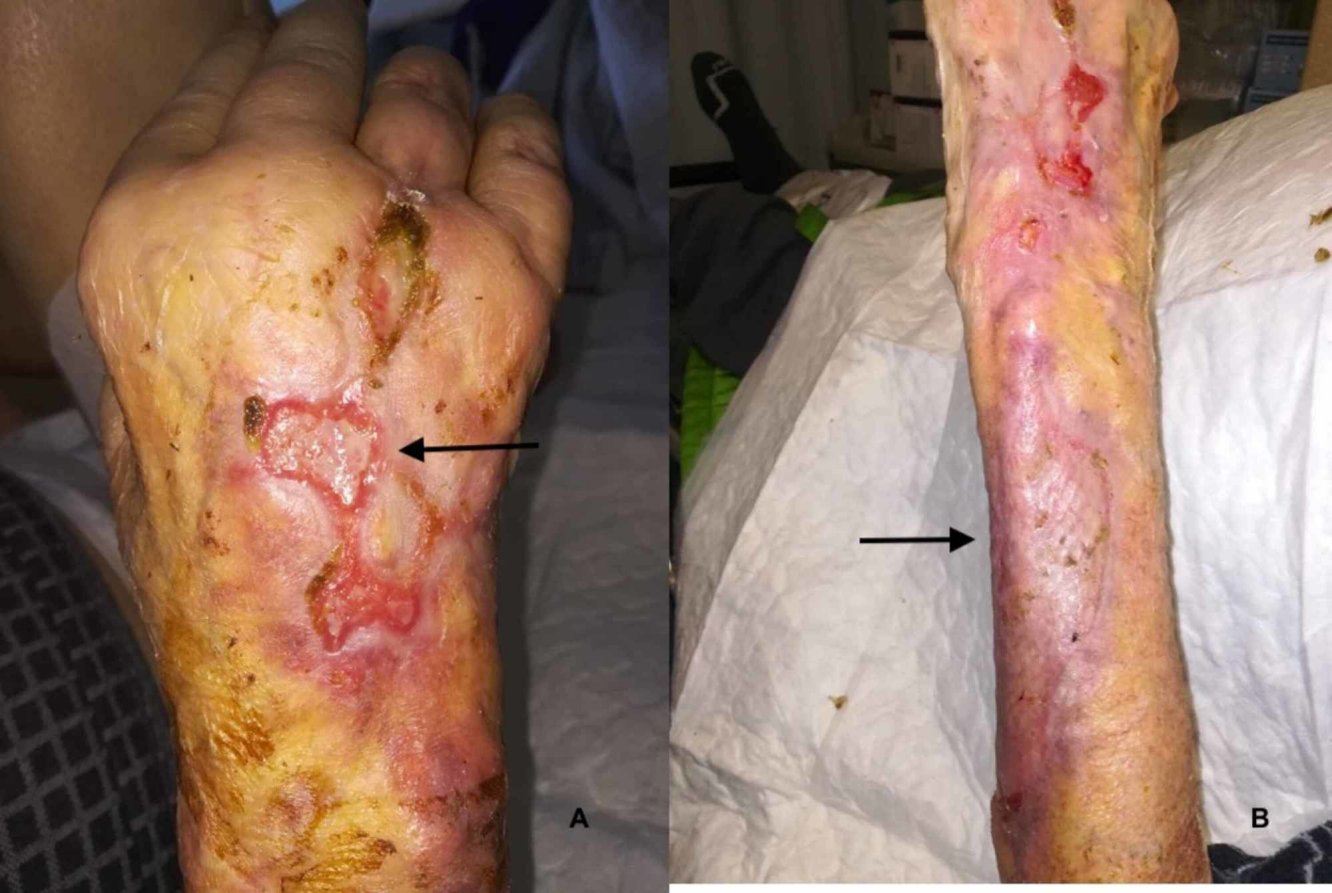
The cutaneous lesion after the skin graft

We observed a rapid improvement of the neurological signs and the healing of the lesion on the left thigh, which had not yet ulcerated. The ulcers on the right arm gradually improved and the patient was transferred to another hospital in order to undergo a skin filling with collagen and then a plastic surgery (Figure 4) with an autologous skin graft (Figure 5). The surgery was successful and the patient was then switched to a maintenance therapy with fluconazole 200 mg from October 2019.

At the beginning of April 2020, during the terrible outbreak of SARS-CoV-2 (severe acute respiratory syndrome coronavirus 2), the patient, who lived in Lombardy, where the largest epidemic outbreak has developed in Italy, died after a respiratory failure. He presented no signs and symptoms of recrudescence of cutaneous and neurological cryptococcosis, but the cryptococcal antigen was still positive.

## Discussion

Up until now, most of the information we have regarding cryptococcosis is based on experience from HIV-positive patients. However, in recent years, thanks to the introduction of an effective antiretroviral therapy, HIV-positive patients have constituted an ever-smaller percentage of the total cases of cryptococcosis [3,4].

In a U.S. cohort study ﻿of 302 patients diagnosed with cryptococcosis, 108 (36%) were HIV-positive, 84 (28%) were transplant patients, and 110 (36%) were NHNT (non-HIV, non-transplant patients) [4]. NHNT patients constitute a heterogeneous group that includes patients ranging from seemingly healthy to heavily immunodepressed [5,6].

Specific studies on this patient population are yet to be conducted due to both the high variability of the patients and their relatively recent increase in number. Recent studies have shown how the clinical presentation and mortality of this group of patients is different from that of HIV-positive patients, to whom most clinicians are accustomed [6]. In fact, these patients most frequently present with extra-meningeal manifestations, of which the most frequent is pulmonary; cases of skin, osteoarticular, and soft tissue infections have been reported. In a recent study of 158 NHNT patients, ﻿14 (9%) had infections other than pulmonary, bloodstream, or central nervous system. These included cultures from skin (n = 4), urine (n = 2), joint aspirates (n =2), ascites (n = 2), lymph node biopsy (n = 2), bone biopsy (n = 1), and ocular fluid (n = 1) [7].

There is no typical cutaneous lesion of cryptococcosis, but skin involvement is typically characterized by various non-specific presentations (e.g., papules, pustules, nodules, abscesses, edema, panniculitis, and ulcers) and can be due to a primary infection or due to a secondary systemic hematogenous spread [8-10]. Therefore, once a diagnosis is established, the infection of the brain and lungs must be excluded [11].

Our patient presented with two large and necrotic ulcers on his right forearm and was treated with broad-spectrum antibiotics. The initial cutaneous swabs detected an unspecified yeast, which was interpreted as a contamination. The association between the failure of different antibiotics and the onset of neurological symptoms suggested a form of encephalitis; therefore, we performed lumbar puncture in order to exclude an opportunistic infection.

The studies cited above and the clinical course of our patient showed that cutaneous symptoms in an immunocompromised patient should always alert about the possibility of an opportunistic infection such as cryptococcosis. The failure of a broad-spectrum course of antibiotics and the onset of neurological signs can be further differentials in the diagnosis.

Furthermore, the immunological studies, such as peripheral blood lymphocyte typing and immunoglobulin tests, could be useful to inform clinical management and suspect an opportunist infection, even in immunosuppressed non-HIV patients.

The domestic trauma, the extensive interval between the onset of the lesions and the first neurological sign (the headache was first noted 50 days following admission), and the isolation of an unspecified yeast on the first skin swab would lead us to think that the port of entry of the infection was the skin. However, the scarce data that are present in the literature and the typically subacute course of a cryptococcal meningeal infection often lead a patient to overlook these neurological symptoms, especially if the patient has multiple co-morbidities. Even if this case is suggestive of the skin being the primary infected organ, we cannot directly assume this.

An important teaching we learnt from this case is the necessity of direct communication and case discussion between the clinician and the microbiologist in the lab for a more careful and thorough interpretation of contaminations versus true infections in the patient sample. This is particularly important if there is a high clinical suspicion of an opportunistic infection.

## Conclusions

The clinical case presented required a considerable expenditure of resources and energy. In fact, in search of a diagnosis, the hospitalization lasted several weeks consisting of the almost daily treatment with advanced dressings as well as the treatment of complications that arose during his stay. In addition, the follow-up was costly due to a long and difficult rehabilitation. The sudden death of this patient from respiratory failure during the coronavirus epidemic has been a further event in the recent weeks showing us the disparity between medical efforts and clinical results, causing us to put into question our work as healthcare professionals.
